# Dataset on the sustainable smart city development in Indonesia

**DOI:** 10.1016/j.dib.2019.104098

**Published:** 2019-06-05

**Authors:** Restu Mahesa, Gatot Yudoko, Yudo Anggoro

**Affiliations:** School of Business and Management, Bandung Institute of Technology, Indonesia

**Keywords:** Smart city, Sustainability, Readiness, Indonesia

## Abstract

Smart city movements are growing all over the world. The undertaking is expected to solve a plethora of problems arising from urbanization. Indonesia is one of the countries who march toward the development of sustainable smart cities. However, before the government can start a smart city project, they need to assess the readiness of each target city. Data in this article illustrate the readiness of six major cities in Indonesia, which are Semarang, Makassar, Jakarta, Samarinda, Medan, and Surabaya. They represent the four biggest islands in Indonesia. The readiness assessment was based on three main elements and six Smart City Pillars taken from Smart City Master Plan Preparation Guidance Book prepared by Ministry of Communication and Information Technology of the Republic of Indonesia. Those elements serve as a checklist to determine the readiness of the cities. Data for qualitative analysis were gathered through interviews and triangulated through secondary sources, such as publication from Statistics Indonesia and the assessment reports. The dataset contains information on the readiness assessment is presented in this article. The indices of the six region's readiness assessment are presented in percentages.

**Table:** Specifications table

Subject area	Management
More specific subject area	Innovation Management
Type of data	Tables and figures
How data was acquired	Data were acquired from In-depth interviews with several Indonesian governmental ministries and observations from the technical guidance from Indonesian Ministry of Communication & Information to smart cities local government
Data format	Raw and analyzed
Experimental factors	Readiness assessment for sustainable smart cities concept implementation in Indonesia. The assessed elements were regional structures, infrastructures, superstructures, and Six Smart City Pillars.
Experimental features	Measurement indicators were adapted from the Smart City Guidelines for Master Plan Preparation by Ministry of Communication and Information Technology of the Republic of Indonesia (2017) [1]
Data source location	1. Medan as Sumatera Island representative 2. Jakarta as Java Island (western region) representative 3. Semarang as Java Island (middle region) representative 4. Surabaya as Java Island (eastern region) representative 5. Samarinda as Kalimantan Island (Borneo) representative 6. Makassar as Sulawesi Island (Celebes) representative
Data accessibility	This article contains all the dataset
Related research article	R. Mahesa, G. Yudoko, and Y. Anggoro, “Platform Ecosystems for Indonesia Smart Cities,” in 2018 International Conference on Computer, Control, Informatics and its Applications (IC3INA), 2018.DOI: 10.1109/IC3INA.2018.8629537[2]

**Table:** 

**Value of the data** • Our dataset provides the features of seventy-five smart cities development in Indonesia and readiness assessment of six major smart cities based on three main elements and six smart city pillars. • The dataset within this article can be used by the scientific community as comparison materials with other data obtained from other cities, regions, or countries. • The dataset will enable the stakeholders to have more understanding about the progress of smart city development in Indonesia and it helps identifying improvement gaps. • Academics and practitioners from all disciplines can employ the detailed indicators as an assessment checklist for other cities. • The data availability will help policymakers in governments to design responsive policies in terms of sustainable smart city development. • Data in this article can also be used by researchers to study the relationships between urban features of smart city (densities, HDI, and GRDP) and readiness level.

## 1 Data

**Fig. 1:**
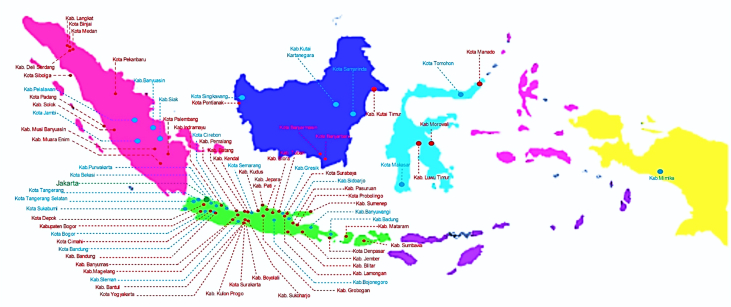
First Phase and Second Phase of Indonesia 100 Smart Cities Movement (Own Elaboration) Note: [Id]: Blue: First phase selected cities/regencies, Red: Second phase selected cities/regencies, Green: Special Region Jakarta, Sources: Ministry of Communication and Information Technology of the Republic of Indonesia [1] (summarized).

Seventy-five cities have been actively engaged in the development of smart city in Indonesia. The group comprises of twenty-four cities that have been selected in the first phase and fifty cities in the second phase. They were a part of the Indonesia 100 Smart Cities Movement initiated by Ministry of Communication and Information Technology of the Republic of Indonesia in 2017 and 2018, and Jakarta as the capital city of Indonesia. The urban features of seventy-five smart cities are presented in Table 1, which provides data on area, population, densities, Human Development Index (HDI), Gross Regional Domestic Product (GRDP), and the ethnic groups. Fig. 1 shows Indonesian map and the location of seventy-five smart cities development.

**Table 1:** Dataset on seventy-five sustainable smart cities development in Indonesia.

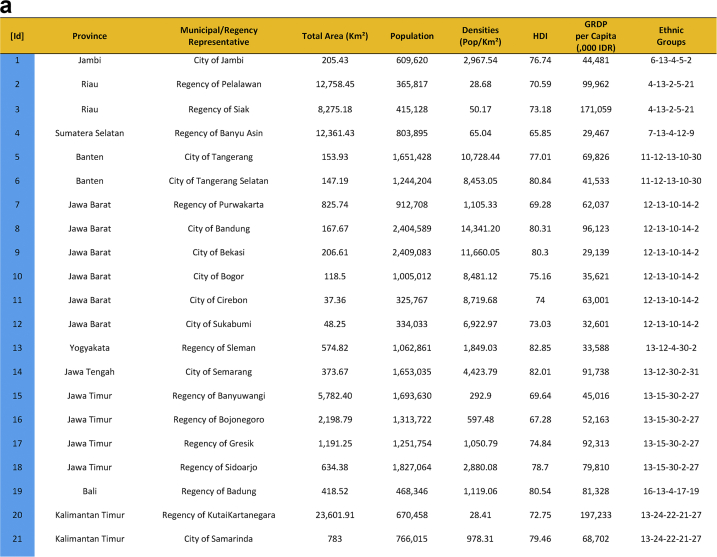 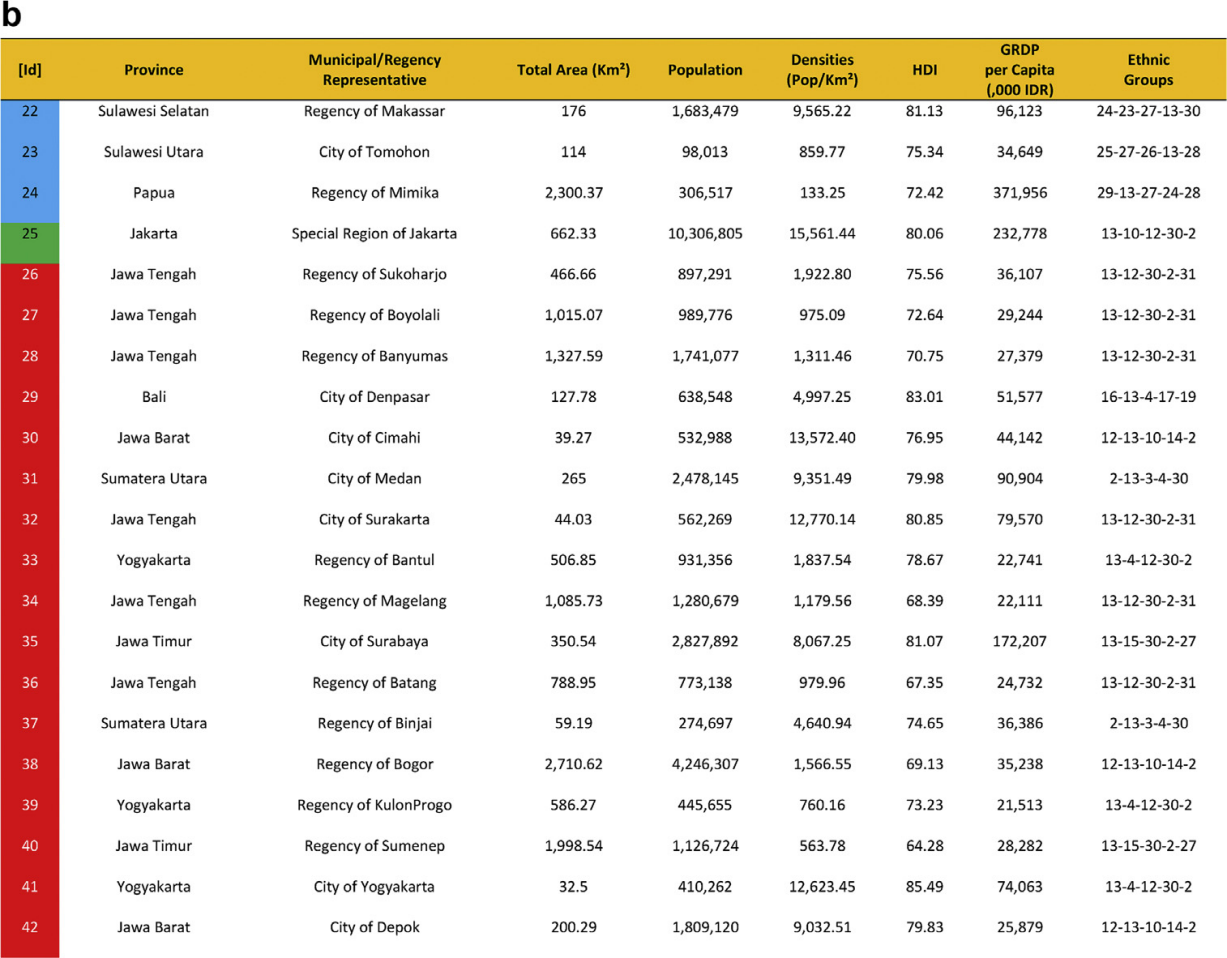 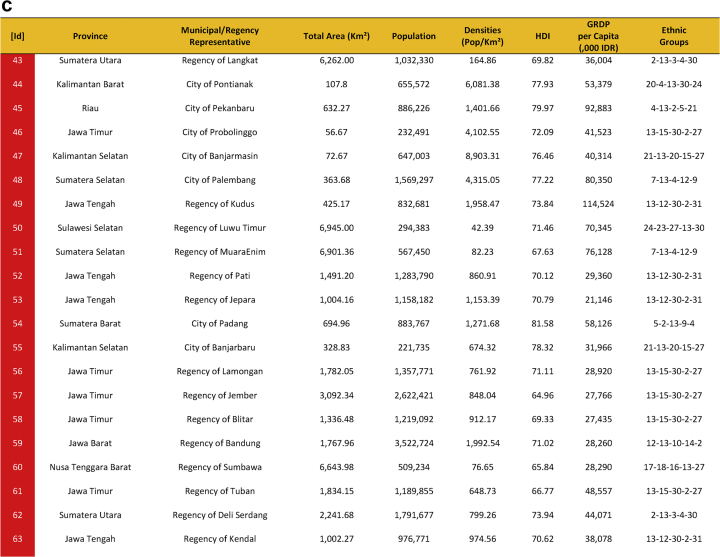 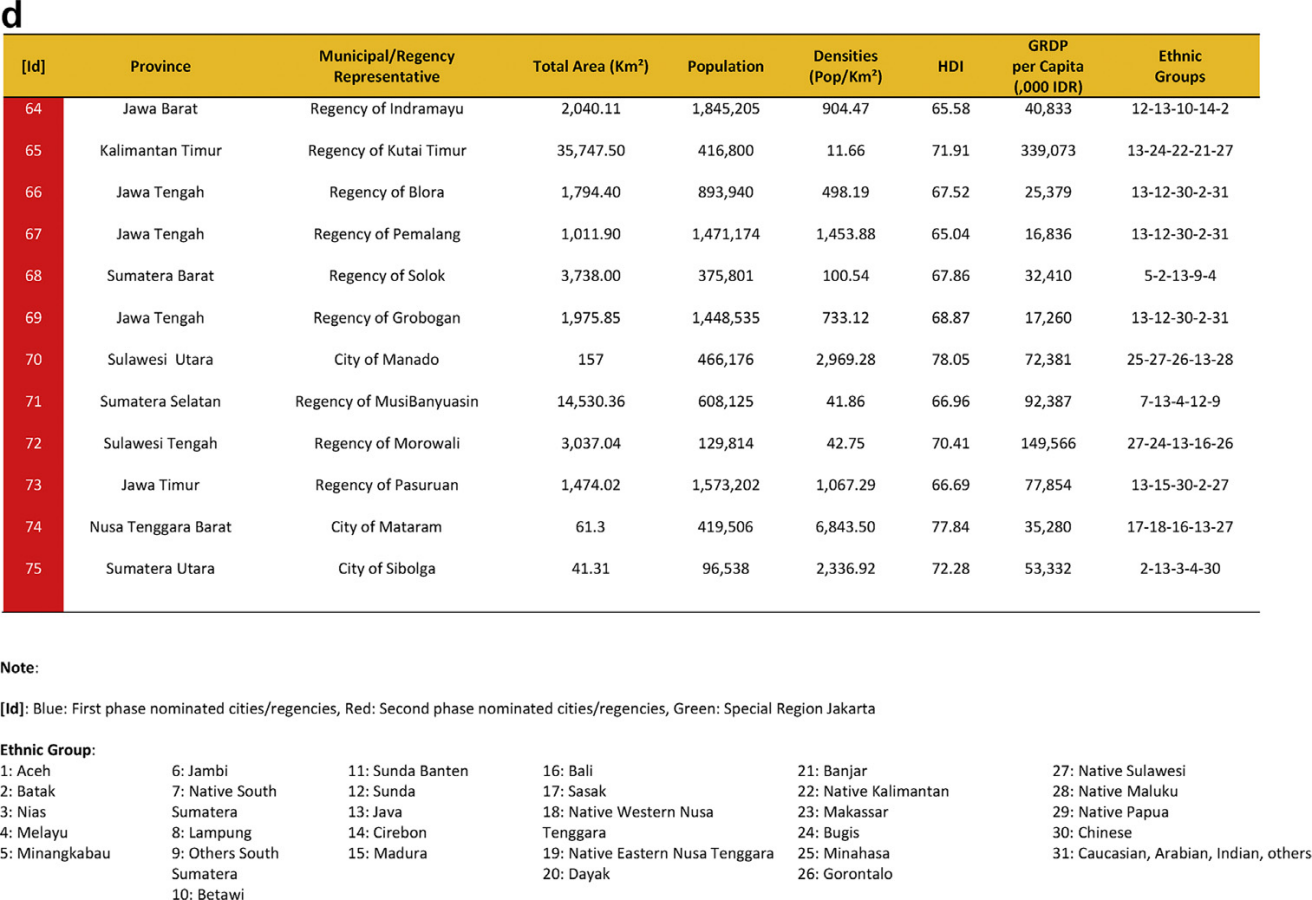

**Fig. 2:**
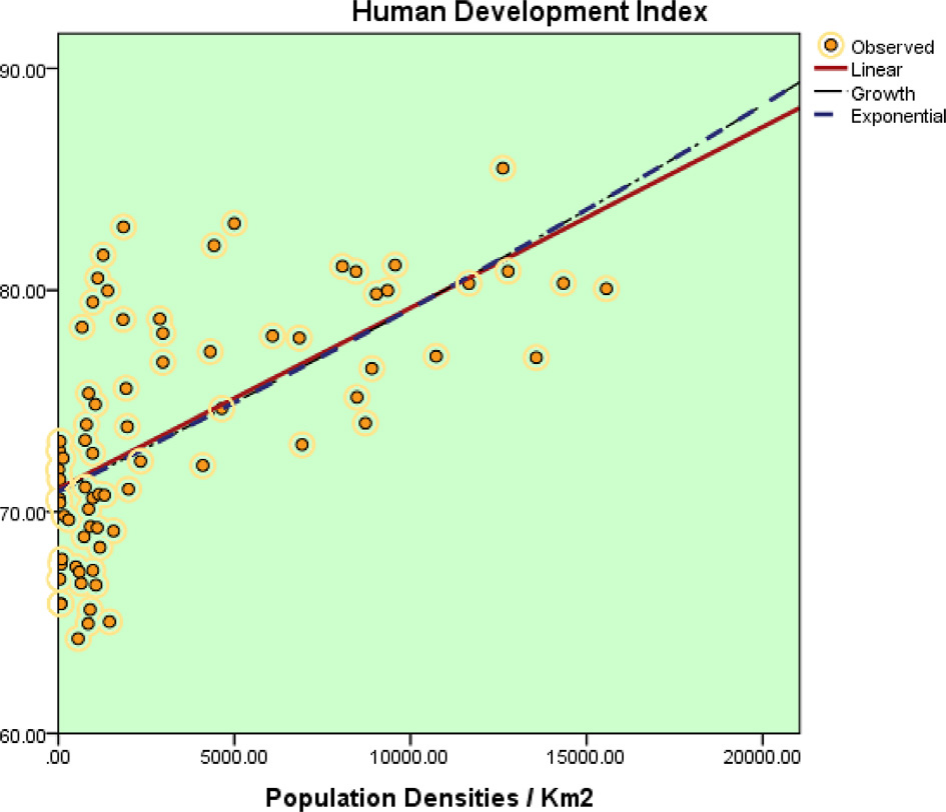
Scatterplot of correlation between densities and HDI on seventy-five selected cities.

**Fig. 3:**
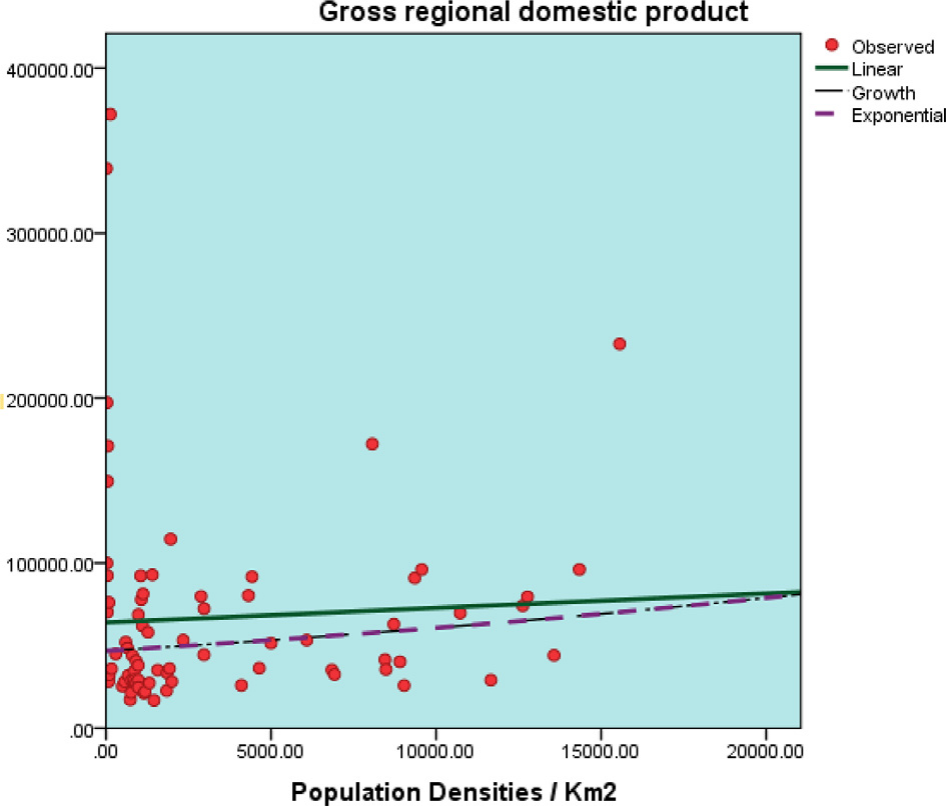
Scatterplot of correlation between densities and GRDP on seventy-five smart cities.

The readiness assessment datasets on six major smart cities are presented in this article. Data measurements were conducted based on three main elements and six smart city pillars. Table 2 shows the example of category assignment on the readiness assessment. The dataset of assessment based on three main elements is presented in Table 3 and assessment based on the six smart city pillars is presented Table 4. The summary of the dataset readiness can be seen in Table 5 and Table 6. Final assessment dataset of smart city index is shown in Table 7. Table 8 shows the correlation between city densities and HDI, while Table 9 demonstrates the correlation between city densities and GRDP. The corresponding scatterplots can be seen in Fig. 2, Fig. 3.

**Table 2:** Example of category assignment.

Components		Regional Readiness to Implement Smart City Concepts Semarang
	**Regional Structure Analysis**	
		*Quality of the Human Capital [HC] – Available/yes = 1, Unavailable/no = 0*
The availability of community that focuses on developing interests in talent, creativity, and culture		1

**Table 3:** Data on Smart Cities three main elements.

No	Components	Regional Readiness to Implement Smart City Concepts					
		Semarang	Makassar	Jakarta	Samarinda	Medan	Surabaya
Regional Structure Analysis							
*Quality of the Human Capital [HC] -- Available/yes = 1, Unavailable/no = 0*							
1	The availability of community that focuses on developing interests in talent, creativity, and culture	1	1	1	1	1	1
2	The existence of software developers' community	1	1	1	1	1	1
3	The existence of digital technology startup or another rising business startup	1	1	1	1	1	1
4	Availability higher education	1	1	1	1	1	1
5	The availability of student Scholarship program from the government	1	1	1	1	1	1
6	Low rates of misdemeanor (minor criminal acts) within region per year	0	0	0	0	0	1
7	Low rates of major criminal acts within region per year	0	0	0	0	0	1
	Subtotal HC [MAX 7]	5	5	5	5	5	7
*Governmental Quality [GQ] -- Available/yes = 1, Unavailable/no = 0*							
1	Staffs with master's or doctoral degrees	1	1	1	1	1	1
2	The availability of highly educated volunteers	1	1	1	1	1	1
3	Staffs with medium to high computer literacy	1	1	1	1	1	1
4	Staffs with medium to high foreign language literacy	1	1	1	1	1	1
5	Broadband access in every public office	1	1	1	1	1	1
6	The availability of LAN/WAN in every public office	1	1	1	1	1	1
7	The availability of Wireless Internet Hotspot in every public office	1	1	1	1	1	1
8	The availability of Independent Data Center services/management	1	1	1	1	1	1
9	The availability of SOP regarding disaster mitigation of governmental data	0	0	0	0	0	0
10	Interoperability of Information Systems regarding regional planning and development	1	1	1	1	1	1
11	Interoperability of Information Systems regarding regional financial management	1	1	1	1	1	1
12	Interoperability of Information Systems regarding virtual governmental office	1	1	1	1	1	1
13	Interoperability of Information Systems regarding regional development monitoring and evaluation	1	1	1	1	1	1
14	Interoperability of Information Systems regarding regional staffing management	1	1	1	1	1	1
15	Interoperability of Information Systems regarding regional legislation management	1	1	1	1	1	1
16	Interoperability of Information Systems regarding regional public services	1	1	1	1	1	1
	Subtotal GQ [MAX 16]	15	15	15	15	15	15
*Regional Financing Capabilities [RFC] Available/yes = 1, Unavailable/no = 0*							
1	Good percentage of local revenue value towards total regional revenue	1	1	1	1	1	1
2	Previous year's budget surplus	0	0	0	1	0	0
3	Good percentage of staffing budget spending towards total regional spending	1	1	1	1	1	1
4	The availability of Smart City Development Program budget allocation per year	1	1	1	1	1	1
5	Local/foreign investment to support regional development	1	1	1	1	1	1
6	The availability of alternative financing resources to support Smart City Development Program	1	1	1	1	1	1
	Subtotal RFC [MAX 6]	5	5	5	6	5	5
Regional Infrastructure Analysis							
*Physical Infrastructure [PI] -- Available/yes = 1, Unavailable/no = 0*							
1	Medium to high percentage of good condition road access	1	1	1	0	0	1
2	Medium to high percentage of good quality pedestrian place	1	1	1	0	0	1
3	Medium to high percentage of good functioning public street lighting	1	1	1	0	0	1
4	Medium to high percentage of good condition road markings	1	1	1	0	1	1
5	Availability of Central Business District	1	1	1	1	1	1
6	Availability of convenient commercial area	1	1	1	1	1	1
7	Availability of education facilities	1	1	1	1	1	1
8	Availability of health facilities	1	1	1	1	1	1
9	Availability of regional security facilities	1	1	1	1	1	1
10	Availability of Water Treatment Plants	1	1	1	1	1	1
11	Availability of Power Plants and Generators	1	1	1	1	1	1
12	Availability of Toll Roads	1	1	1	1	1	1
13	Availability of Train Railways	1	1	1	0	1	1
14	Availability of industrial, manufacturing, inventory, and or logistic management zone	1	1	1	1	1	1
15	Availability of Seaports	1	1	1	1	1	1
16	Availability of Airports	1	1	1	1	1	1
	Subtotal PI [MAX 16]	16	16	16	11	13	16
*Digital Infrastructure [DI] -- Available/yes = 1, Unavailable/no = 0*							
1	Availability of broadband area with 4G or 3G connectivity	1	1	1	1	1	1
2	Availability of affordable broadband internet access for citizen	1	1	1	1	1	1
3	Availability of stable and medium to high internet access for daily activities	1	1	1	1	1	1
4	Good percentage of household's electricity	1	1	1	1	1	1
5	Low rate occurrences of power outage	1	1	1	1	1	1
6	Good percentage of school with internet connectivity	1	1	1	1	1	1
7	Good percentage of health facilities using electronic system services	1	1	1	1	1	1
	Subtotal DI [MAX 7]	7	7	7	7	7	7
*Social Infrastructure [SI] -- Available/yes = 1, Unavailable/no = 0*							
1	Availability of learning facility at the Kelurahan level	1	1	1	1	1	1
2	Availability of open public area at the housing complex	1	1	1	1	1	1
3	Availability of open public hall at the Kelurahan level	1	1	1	1	1	1
4	Availability of sport center facility at the Kelurahan level	1	1	1	1	1	1
5	Availability of open public library in within region	1	1	1	1	1	1
	Subtotal SI [MAX 5]	5	5	5	5	5	5
							
Regional Superstructures Analysis							
*Regional Policies [RP] -- Available/yes = 1, Unavailable/no = 0*							
1	Availability of Regulation regarding Regional Smart City Council	1	1	0	1	1	0
2	Availability of Regional leader regulation regarding Smart City Executives	1	1	0	1	1	0
3	Availability of Regional Smart City Master plan	1	1	1	1	1	0
4	Availability of Regulation regarding regional Smart City Master plan	1	1	0	1	1	0
5	Smart City vision and mission are aligned with regional development focus	1	1	1	1	1	0
6	Certainty regarding the sustainability of Smart City Program for long-term period	1	1	1	1	0	0
7	Existence of evaluation and appreciation mechanism toward the staff performance on Smart City implementation program.	1	1	1	1	1	0
	Subtotal RP [MAX 7]	7	7	4	7	6	0
*Regional Institution Readiness [RIR] -- Available/yes = 1, Unavailable/no = 0*							
1	Existence of Regional Smart City Council	1	1	0	1	1	0
2	Existence of Smart City Executives	1	1	0	1	1	0
3	Availability of standard operation procedure regarding Smart City	1	0	0	0	0	0
4	Existence of smart City governance team in every Organisasi Perangkat Daerah	1	1	0	1	1	0
	Subtotal RIR [MAX 4]	4	3	0	3	2	0
							
*Regional Community Organization [RCO] -- Available/yes = 1, Unavailable/no = 0*							
1	Existence of Community Service Institution at local University	1	1	1	1	1	1
2	Availability of community organization that supports Smart City program within region	1	0	1	0	0	1
3	Medium to Good percentage regarding amount of community organization supporting Smart City program	1	0	1	0	0	1
4	Availability of Government operational support towards Smart City based community organization	1	0	1	0	0	1
5	Medium to Good percentage regarding amount of Smart City based community organization which already has definitive secretariat	1	0	1	0	0	1
6	Existence of Expert participation from the local university supporting regional Smart City council	1	1	1	1	1	1
	Subtotal RCO [MAX 6]	6	2	6	2	2	6

**Table 4:** Data on smart city pillars.

No	Components	Semarang		Makassar		Jakarta		Samarinda		Medan		Surabaya	
		MP	Act	MP	Act	MP	Act	MP	Act	MP	Act	MP	Act
Smart Governance													
*Public Services [PSV] -- If Eligible = 1 Point per indicator, Unknown = 0 Point*													
1	Existence of Public administration services	1	1	1	1	1	1	1	1	1	1	0	1
2	Improvement for basic commodities supply services and facilities	1	1	1	1	1	1	1	1	1	1	0	1
3	Improvement for basic needs of physical and digital infrastructure	1	1	1	1	1	1	1	1	1	1	0	1
	Subtotal PSV [MAX 6]	6		6		6		6		6		3	
*Efficient Bureaucracy Management [EBM] -- If Eligible = 1 Point per indicator, Unknown = 0 Point*													
1	Bureaucracy management based on fairness, accountability, and transparency principles (such as: e-Planning and e-Budgeting)	1	1	1	1	1	1	1	1	1	1	0	1
	Subtotal EBM [MAX 2]	2		2		2		2		2		1	
*Efficient Public Policy [EPP] -- If Eligible = 1 Point per indicator, Unknown = 0 Point*													
1	Policy making based on positive impact towards society by conducting interactive communication with them	1	1	1	1	1	1	1	1	1	1	0	1
2	Easy access to Government Regulation Information System	1	1	1	1	1	1	1	1	1	1	0	1
	Subtotal EPP [MAX 4]	4		4		4		4		4		2	
Smart Branding													
*Regional Tourism Branding [RTB] -- If Eligible = 1 Point per indicator, Unknown = 0 Point*													
1	Build and develop decent tourism destinations for tourists	1	1	1	1	1	1	1	1	1	1	0	1
2	Build and develop decent tourism infrastructure and facilities	1	1	1	1	1	1	1	1	1	1	0	1
3	Build hospitality culture including foreign language proficiency for the tour guide and similar activities	1	1	1	1	1	1	1	1	1	1	0	1
	Subtotal RTB [MAX 6]	6		6		6		6		6		3	
*Regional Business Branding [RBB] -- If Eligible = 1 Point per indicator, Unknown = 0 Point*													
1	Build platforms to promote conducive and comfortable of commercial ecosystems (such as regional e-Marketplace)	1	1	1	1	1	1	1	1	0	0	0	1
2	Creating investment friendly ecosystems for regional development (such as investment portal, lounge, forum)	1	1	1	1	1	1	1	0	1	1	0	1
3	Promoting and monetizing regional creative industries	1	1	1	1	1	1	1	0	1	1	0	1
	Subtotal RBB [MAX 6]	6		6		6		4		4		3	
*Regional Image Branding [RIB] -- If Eligible = 1 Point per indicator, Unknown = 0 Point*													
1	Rearrangement and revitalization of regional architectural values based on local wisdom and similar aspects	1	1	1	1	1	1	1	1	0	0	0	1
2	Build well-organized regional planning and design	1	1	0	0	1	1	0	0	0	0	0	1
	Subtotal RIB [MAX 4]	4		2		4		2		0		2	
Smart Economy													
*Creating Competitive Industrial Ecosystems [CCIE] -- If Eligible = 1 Point per indicator, Unknown = 0 Point*													
1	Creating regional competitive industrial ecosystems especially focused on integration of leading, secondary, and tertiary market sectors such as agriculture, fisheries, and farm with manufacturing, food processing and regional marketplaces	1	1	1	1	0	0	1	1	0	0	0	1
	Subtotal CCIE [MAX 2]	2		2		0		2		0		1	
*Welfare [We] -- 5 Points per indicator, Unknown = 0 Point*													
1	Developing community welfare program through the domestic productivity income	1	1	1	1	1	1	1	1	1	1	0	1
2	Developing regional employment program	1	1	1	1	1	1	1	1	1	1	0	1
3	Creating integrated conventional and digital economy program	1	1	0	0	1	1	1	1	0	0	0	1
	Subtotal We [MAX 6]	6		4		6		6		4		3	
Smart Living													
*Harmonization of Regional Layout [HRL] -- If Eligible = 1 Point per indicator, Unknown = 0 Point*													
1	Creating comfortable and harmonious living environment	1	1	1	1	1	1	1	1	1	1	0	1
	Subtotal HRL [MAX 2]	2		2		2		2		2		1	
*Health Facilities [HF] -- If Eligible = 1 Point per indicator, Unknown = 0 Point*													
1	Creating reliable access to the availability of healthy foods, healthcare facilities, and sport center facilities.	1	1	1	1	1	1	1	1	1	1	0	1
	Subtotal HF [MAX 2]	2		2		2		2		2		1	
*Mobility Transport [MT] -- If Eligible = 1 Point per indicator, Unknown = 0 Point*													
1	Creating environmentally friendly transportation ecosystems that connecting public needs	1	1	1	1	1	1	1	1	1	1	0	1
	Subtotal MT [MAX 2]	2		2		2		2		2		1	
Smart Society													
*Efficient Public Interactivity [EPI] -- If Eligible = 1 Point per indicator, Unknown = 0 Point*													
1	Creating public ecosystems to support regional Smart City program	1	1	1	1	1	1	1	1	1	1	0	1
2	Community development program to gain public productivity and positive behavior	1	1	1	1	1	1	1	1	1	1	0	1
	Subtotal EPI [MAX 4]	4		4		4		4		4		2	
*Learning Ecosystems [LE] -- If Eligible = 1 Point per indicator, Unknown = 0 Point*													
1	Creating educational ecosystems which can support formal and non-formal learning systems	1	1	1	1	1	1	1	1	1	1	0	1
2	Building affordable educational platform for the society	1	1	1	1	1	1	1	1	1	1	0	1
	Subtotal LE [MAX 4]	4		4		4		4		4		2	
*Public Security [PSC] -- If Eligible = 1 Point per indicator, Unknown = 0 Point*													
1	Creating Security Management System for public safety and security	1	1	1	1	1	1	1	1	1	1	0	1
	Subtotal PSC [MAX 2]	2		2		2		2		2		1	
Smart Environment													
*Environment Protection [EP] -- If Eligible = 1 Point per indicator, Unknown = 0 Point*													
1	Developing integrated monitoring and reporting protection systems for land, water, and air	1	1	1	1	1	1	1	1	1	1	0	1
2	Build smart green open space	1	1	1	1	1	1	1	1	1	1	0	1
3	Restoration of polluted rivers and watersheds	1	1	1	1	1	1	1	1	1	1	0	1
4	Controlling air pollution	1	1	1	1	1	1	1	1	1	1	0	1
	Subtotal EP [MAX 8]	8		8		8		8		8		4	
*Waste Management [WM] -- If Eligible = 1 Point per indicator, Unknown = 0 Point*													
1	Developing household and public waste management system	1	1	1	1	1	1	1	0	0	0	0	1
2	Developing industrial waste management system	1	1	1	1	1	1	1	0	0	0	0	1
3	Protection for ecosystem stability	1	1	1	1	1	1	1	0	0	0	0	1
	Subtotal WM [MAX 6]	6		6		6		3		0		3	
*Energy Management [EM] -- If Eligible = 1 Point per indicator, Unknown = 0 Point*													
1	Efficient and responsible energy utilization program	1	1	1	1	1	1	1	1	1	1	0	1
2	Alternative energy development program based on sustainable and environmentally friendly principles	1	1	1	1	1	1	1	1	1	1	0	1
	Subtotal EM [MAX 4]	4		4		4		4		4		2	

Note: MP = Master Plan, Act = Actual (real condition).

**Table 5:** Data summary of Smart Cities Main Elements.

	Semarang	Makassar	Jakarta	Samarinda	Medan	Surabaya
Subtotal HC	5	5	5	5	5	7
Subtotal GQ	15	15	15	15	15	15
Subtotal RFC	5	5	5	6	5	5
Total Reg. Structure [MAX 29]	25	25	25	26	25	27
Subtotal PI	16	16	16	11	13	16
Subtotal DI	7	7	7	7	7	7
Subtotal SI	5	5	5	5	5	5
Total Reg. Infrastructure [MAX 28]	28	28	28	23	25	28
Subtotal RP	7	7	4	7	6	0
Subtotal RIR	4	3	0	3	2	0
Subtotal RCO	6	2	6	2	2	6
Total Reg. Superstructures [MAX 17]	17	12	10	12	10	6
Total S + I + S = Max 74	70	65	63	61	60	61

**Table 6:** Data summary of Smart City Pillars.

	Semarang	Makassar	Jakarta	Samarinda	Medan	Surabaya
Subtotal PSV	6	6	6	6	6	3
Subtotal EBM	2	2	2	2	2	1
Subtotal EPP	4	4	4	4	4	2
Total Smart Governance [MAX 12]	12	12	12	12	12	6
Subtotal RTB	6	6	6	6	6	3
Subtotal RBB	6	6	6	4	4	3
Subtotal RIB	4	2	4	2	0	2
Total Smart Branding [MAX 16]	16	14	16	12	10	8
Subtotal CCIE	2	2	0	2	0	1
Subtotal We	6	4	6	6	4	3
Total Smart Economy [MAX 8]	8	6	6	8	4	4
Subtotal HRL	2	2	2	2	2	1
Subtotal HF	2	2	2	2	2	1
Subtotal MT	2	2	2	2	2	1
Total Smart Living [MAX 6]	6	6	6	6	6	3
Subtotal EPI	4	4	4	4	4	2
Subtotal LE	4	4	4	4	4	2
Subtotal PSC	2	2	2	2	2	1
Total Smart Society [MAX 10]	10	10	10	10	10	5
Subtotal EP	8	8	8	8	8	4
Subtotal WM	6	6	6	3	0	3
Subtotal EM	4	4	4	4	4	2
Total Smart Environment [MAX 18]	18	18	18	15	12	9
Total Smart G + B + E + L + S + E = Max 70	70	66	68	63	54	35

**Table 7:** Data summary on sustainable smart cities development in Indonesia.

	Semarang	Makassar	Jakarta	Samarinda	Medan	Surabaya
TOTAL 3Main Elements [3ME] [Max 74]	70	65	63	61	60	61
TOTAL Smart City Pillars [SCP] [Max 70]	70	66	68	63	54	35
Total 3ME + SCP = 144	140	131	131	124	114	96
Regional Readiness [144 × 100%]	97%	91%	91%	86%	79%	66%

**Table 8:** Correlation between densities and HDI for seventy-five smart cities.

Descriptive Statistics	Mean	Std. Deviation	N
Population Densities/Km2	3387.2621	4161.49056	75
Human Development Index	73.7996	5.40077	75
Correlations		Population Densities/Km2	Human Development Index
Population Densities/Km2	Pearson Correlation	1	.629^a^
	Sig. (2-Tailed)		.000
	N	75	75
Human Development Index	Pearson Correlation	.629^a^	
	Sig. (2-Tailed)	.000	
	N	75	75

Linear					
**Model Summary**					
R	R Square	Adjusted R Square	Std. Error of The Estimate		
.629	.395	.387	4.228		
**ANOVA**					
	Sum of Squares	df	Mean Square	F	Sig.
Regression	853.524	1	853.524	47.748	.000
Residual	1304.928	73	17.876		
Total	2158.451	74			
**Coefficients**					
	Unstandardized B	Coeff. Std. Error	Standardized Coeff. Beta	t	Sig.
Densities	.001	.000	.629	6.910	.000
(Constant)	71.035	.631		112.544	.000
Growth					
**Model Summary**					
R	R Square	Adjusted R Square	Std. Error of The Estimate		
.625	.391	.382	.058		
**ANOVA**					
	Sum of Squares	df	Mean Square	F	Sig.
Regression	.155	1	.155	46.805	.000
Residual	.241	73	.003		
Total	.396	74			
**Coefficients**					
	Unstandardized B	Coeff. Std. Error	Standardized Coeff. Beta	t	Sig.
Densities	1.099E-5	.000	.625	6.841	.000
(Constant)	4.261	.009		496.337	.000
Exponential					
**Model Summary**					
R	R Square	Adjusted R Square	Std. Error of The Estimate		
.625	.391	.382	.058		
**ANOVA**					
	Sum of Squares	df	Mean Square	F	Sig.
Regression	.155	1	.155	46.805	.000
Residual	.241	73	.003		
Total	.396	74			
**Coefficients**					
	Unstandardized B	Coeff. Std. Error	Standardized Coeff. Beta	t	Sig.
Densities	1.009E-5	.000	.625	6.841	.000
(Constant)	70.915	.609		116.471	.000

a Correlation is significant at the 0.01 level (2-Tailed).

**Table 9:** Correlation between densities and GRDP for seventy-five smart cities.

Descriptive Statistics	Mean	Std. Deviation	N
Population Densities/Km2	3387.262	4161.49	75
Gross Regional Domestic Product	67050.38	64045.57	75
Correlations		Population Densities/Km2	Gross Regional Domestic Product
Population Densities/Km2	Pearson Correlation	1	.057
	Sig. (2-Tailed)		.630
	N	75	75
Gross Regional Domestic Product	Pearson Correlation	.057	1
	Sig. (2-Tailed)	.630	
	N	75	75

Linear					
**Model Summary**					
R	R Square	Adjusted R Square	Std. Error of The Estimate		
.057	.003	-.010	64379.442		
**ANOVA**					
	Sum of Squares	df	Mean Square	F	Sig.
Regression	971792032.	1	971792032.2	.234	.630
Residual	3.026E+11	73	4144712591		
Total	3.035E+11	74			
**Coefficients**					
	Unstandardized B	Coeff. Std. Error	Standardized Coeff. Beta	t	Sig.
Densities	.871	1.798	.057	.484	.630
(Constant)	64100.735	9610.952		6.670	.000
Growth					
**Model Summary**					
R	R Square	Adjusted R Square	Std. Error of The Estimate		
.159	.025	.012	.676		
**ANOVA**					
	Sum of Squares	df	Mean Square	F	Sig.
Regression	.866	1	.866	1.897	.173
Residual	33.330	73	.457		
Total	34.196	74			
**Coefficients**					
	Unstandardized B	Coeff. Std. Error	Standardized Coeff. Beta	t	Sig.
Densities	2.600E-5	.000	.159	1.377	.173
(Constant)	10.756	.101		106.624	.000
Exponential					
**Model Summary**					
R	R Square	Adjusted R Square	Std. Error of The Estimate		
.159	.025	.012	.676		
**ANOVA**					
	Sum of Squares	df	Mean Square	F	Sig.
Regression	.866	1	.866	1.897	.173
Residual	33.330	73	.457		
Total	34.196	74			
**Coefficients**					
	Unstandardized B	Coeff. Std. Error	Standardized Coeff. Beta	t	Sig.
Densities	2.600E-5	.000	.159	1.377	.173
(Constant)	46887.466	4729.671		9.913	.000

### 1.1 Regional readiness measures

The essence of the Smart City concept is the city and all its components can manage existing resources to support and maintain the continuity of the ecosystems. Two steps were used to assess regional readiness. First step, the regions were assessed on three main elements based on the Smart City Master Plan Preparation Guidance Book [1]. The elements used are:

1. Structures including human capital, financial capital, and governance capital.
2. Infrastructures including physical, digital, and social.
3. Superstructures including regional regulation (statute), institutional, and implementation development based on smart-city aspects.

Second step, the cities were further assessed based on the dimensions of six Smart City Pillars. The measures used are:

1. Smart Governance including Public Service, Bureaucracy, and Public Policy
2. Smart Branding including Tourism Branding, Business Branding, and City Appearance Branding
3. Smart Economy including Competitive Industry, Welfare, and Transaction
4. Smart Living including Harmonization of Regional Layout, Health Facilities, Mobility Access
5. Smart Society including Community Interaction, Learning Ecosystem, and Safety & Security
6. Smart Environment including Environmental Protection, Waste Management, and Energy Responsibility

## 2 Experimental design, materials, and methods

### 2.1 Samples

The dataset in this article relates to the concept of Indonesia Smart Cities Platform Ecosystems which discussed the effort of the Indonesian government to implement the Smart City concept in all national development aspects [2]. This paper has a mission to present a dataset of the readiness assessment of smart cities chosen by the Ministry of Communication and Information Technology of The Republic of Indonesia for the 100 Smart City program. The Ministry, up until this article is written, has held two selection phases since 2017. They have chosen seventy-four cities (see Table 1). The selection process was expected to be fully accomplished in late 2019 or early 2020. The Ministry's panelists which consisted of academics, private sectors, and members of local/central governments were required to adhere to the Smart City Master Plan Preparation Guidance Book.

This dataset has six major cities (i.e. samples) that represent Indonesia's main islands, which are Medan, Jakarta, Semarang, Surabaya, Samarinda, and Makassar. The Ministry did not include Jakarta in their 100 Smart City Program because the city is a Special Region which does not belong to West Java, Central Java, or East Java province. However, Jakarta was selected as sample because this city, along with Bandung and Surabaya, is a pioneer in the development of smart cities in Indonesia and its effort towards sustainable smart cities has not ceased.

The six major cities were also chosen because of the availability of regional logistical support facilities and infrastructure, such as airports, seaports, container terminals, warehousing, and access to the main road. The availability of seaport became one of the most important considerations because seaport has roles that cannot be replaced by other modes, such as airports, highways, and trains [2]. Seaport has essential functions because Indonesia is an archipelago. The unavailability of a seaport was the reason why this study excluded several cities like Bandung and Bogor.

### 2.2 Data gathering and analysis

The qualitative data were gathered through a series of interviews. To ensure the validity of the data, the interviewers questioned members of the central governments and academics. Each interview was transcribed. Content analysis was done to interpret and code textual material (i.e., raw texts from interviews transcriptions). The data in the form of interview quotes were then categorized based on the pre-determined theme and theoretical constructs (i.e., the three main elements and the six smart city pillars). The data were further corroborated using secondary data, such as publications from Statistics Indonesia, the assessment results, end-of-year performance reports form the central and local governments, and New Urban Agenda from Ministry of Public Works and Housing.

In accordance to Yin's recommendation [3], the qualitative data in this research were quantified to obtain sample's regional readiness. After procuring data from interviews and secondary sources, points were assigned to those answers. For example (see Table 2), one of the readiness indicators is “the availability of community that focuses on developing interests in talent, creativity, and culture.” The interviewees both indicated that such community existed in Semarang, and the secondary data verified it. Thus, Semarang is given “1” point under this specific indicator.

After gathering data for all sample cities, the readiness of each city was calculated by using an equation (see Eq.1) based on works of Atmojo et al. [4] and Chang and Huang [5].

(Eq.1) $$Regional_{Readiness}=\frac{\sum_{i}^{j}3MainElements+\sum_{i}^{j}SmartCityPillars}{\sum Max\left(3MainElements+SmarCityPillars\right)}x100\%$$

Eq. (1) generated the benchmarked regional readiness dataset of six cities in percentages. In this dataset, the regional readiness is the ratio between a city's total score on three main elements and six Smart City Pillars and the maximum point a city can reach (i.e., 144).

## Appendix Interview questions

• Questions related to three main elements of Smart City
  1. What kind of regional structures are needed for the development of smart city?
  2. What kind of regional infrastructures are needed for the development of smart city?
  3. What kind of regional superstructures are needed for the development of smart city?
• Questions related to six Smart City Pillars
  1. What are the most important aspects of smart governance which must be considered to develop smart city?
  2. What are the most important aspects of smart branding which must be considered to develop smart city?
  3. What are the most important aspects of smart economy which must be considered to develop smart city?
  4. What are the most important aspects of smart living which must be considered to develop smart city?
  5. What are the most important aspects of smart transport which must be considered to develop smart city?
  6. What are the most important aspects of smart environment which must be considered to develop smart city?

## References

[1] (2017). Ministry of Communication and Information Technology of the Republic of Indonesia, “Guidelines for Smart City Master Plan - towards 100 Smart Cities Movement,”.

[2] Mahesa R., Yudoko G., Anggoro Y. (2018). Platform Ecosystems for Indonesia Smart Cities. 2018 International Conference on Computer, Control, Informatics and its Applications (IC3INA).

[3] Yin R.K. (2014). Case Study Research : Design and Methods.

[4] Atmojo R.N.P., Anindito, Pardamean B., Abbas B.S., Cahyani A.D., Manulang I.D. (2014). Fuzzy simple additive weighting based, decision support system application for alternative confusion reduction strategy in smartphone purchases. Am. J. Appl. Sci..

[5] Chang H.H., Huang W.C. (2006). Application of a quantification SWOT analytical method. Math. Comput. Model..

